# Designing and testing scene enhancement algorithms for patients with retina degenerative disorders

**DOI:** 10.1186/1475-925X-9-27

**Published:** 2010-06-18

**Authors:** Walid I Al-Atabany, Muhammad A Memon, Susan M Downes, Patrick A Degenaar

**Affiliations:** 1 Institute of Biomedical Engineering, Imperial College, South Kensington, London, SW7 1LU, UK; 2 Department of Neuroscience, Imperial College, South Kensington, London, SW7 1LU, UK; 3 Oxford Eye Hospital, Headley Way, Oxford, OX3 9DU, UK; 4 Department of Biomedical Engineering, Helwan University, Cairo, 11421, Egypt

## Abstract

**Background:**

Retina degenerative disorders represent the primary cause of blindness in UK and in the developed world. In particular, Age Related Macular Degeneration (AMD) and Retina Pigmentosa (RP) diseases are of interest to this study. We have therefore created new image processing algorithms for enhancing the visual scenes for them.

**Methods:**

In this paper we present three novel image enhancement techniques aimed at enhancing the remaining visual information for patients suffering from retina dystrophies. Currently, the only effective way to test novel technology for visual enhancement is to undergo testing on large numbers of patients. To test our techniques, we have therefore built a retinal image processing model and compared the results to data from patient testing. In particular we focus on the ability of our image processing techniques to achieve improved face detection and enhanced edge perception.

**Results:**

Results from our model are compared to actual data obtained from testing the performance of these algorithms on 27 patients with an average visual acuity of 0.63 and an average contrast sensitivity of 1.22. Results show that *Tinted Reduced Outlined Nature (TRON) *and *Edge Overlaying *algorithms are most beneficial for dynamic scenes such as motion detection. *Image Cartoonization *was most beneficial for spatial feature detection such as face detection. Patient's stated that they would most like to see *Cartoonized *images for use in daily life.

**Conclusions:**

Results obtained from our retinal model and from patients show that there is potential for these image processing techniques to improve visual function amongst the visually impaired community. In addition our methodology using face detection and efficiency of perceived edges in determining potential benefit derived from different image enhancement algorithms could also prove to be useful in quantitatively assessing algorithms in future studies.

## Background

There are thought to be 38 million people suffering from blindness worldwide [1], and this number is expected to double over the next 25 years. Additionally, there are 110 million people who have severely impaired vision. The low vision pathologies of this latter group can be divided mainly into two categories; those that predominantly suffer from a loss of visual acuity due to macular degenerations, and those that predominantly suffer from a reduction in the overall visual field such as Retinitis Pigmentosa. In many countries, there is an increasing prevalence of diabetic retinopathy and an ageing population with 1 in 3 over the age of 75 being affected with some form of AMD [2].

Despite advances in treatment such as antivascular endothelial growth factor agents for exudative age related macular degeneration (wet-AMD) and medical and surgical management of glaucoma, there are still a significant number of conditions which lead to severe sight loss. Dry AMD, and untreatable diabetic retinopathy, as well as inherited retinal degenerations such as Retinitis Pigmentosa (RP) are significant examples of these [3].

People with visual acuity impairment suffer from a range of problems affecting their mobility and quality of life [4].

Electronically enhanced visual aids have been proposed which offer a number of distinct advantages over conventional low vision aids in low vision rehabilitation [5]. Prothero [6] overlaid virtual cues on the real scene, to improved the mobility of patients with Akinesia. Massof and Rickman [7] developed a low vision imaging system ("ELVIS") at Johns Hopkins University, which mainly provides magnification and contrast enhancement. Wolffsohn [8] overlaid edges on the original scenes, to enhance the television viewing. Although the results were good, using the Gaussian filter as a scene pre-smoothing before extracting the edges blurs the important features as well as the irrelevant textures.

The Harvard Vision Rehabilitation Lab group has published numerous papers in the field of vision rehabilitation. They multiplexed minified edges over the original scene on a see-through display [9]. However, there is the potential for inattentional blindness, which is the inability of observers to maintain awareness of events in more than one of two superimposed scenes. Apfelbaum [10], tested the effect of vision-multiplexing in reducing the inattentional blindness phenomena, but he found that it does not have any positive or negative effect on reducing the inattentional blindness. Also, Fullerton et al [11] and Peli et al [12,13] have tackled the problem of enhancing television images by overlaying extracted edges on the original images. However, patients reported some inconvenience due to the appearance of randomly highlighted pixels which was due to enhancing the noise as well as the major objects. Fernando [14], recently developed a portable aiding system by applying a digital zooming and edge enhancement to the scene, especially aimed at patients with RP.

Most of the work described above has been based on two main techniques; image resizing and edge overlay. However, both approaches rely on edge extraction techniques which can amplify irrelevant information such as noise or textural detail in addition to significant features. Everingham [15] tackled the irrelevant information enhancement problem by classifying the objects in the scene into eight main colored objects. Classification allowed separation of those objects from irrelevant details. The limitation is that the scene can only be separated on the basis of eight pre-defined objects with losing the ability to see the natural color information of the visual scene.

Image enhancement and segmentation algorithms have been progressively developed in the field of medical image processing [16-19]. However, scalability and implementability of these algorithms on portable and low power consumption devices is our main concern [20].

From the literature it is clear that there is a lack of objective assessment tools to quantitatively evaluate novel image enhancement methods, unless testing them on large numbers of patients. In this paper we describe three image enhancement techniques developed for patients with low vision due to retinal degeneration. To test these techniques, we have built a retinal image processing model of the degenerate retina to assess the degradation of the visual information. In our model, we aim to understand what information is transferred to the visual cortex rather than assess detailed low-level synaptic processing. Thus, our model aims to replicate the main centre-surround and color opponent spatial information processing tasks of the retina. We then reconstruct the image to assess loss of information and the impact of any visual defects. Using our mode, we create a virtual scotomata and asses its impact on the original and enhanced scenes. In order to form quantitative, we have used face detection as a key visual task. Using this it is possible to assess the effect of different image enhancements for different types and severity of retinal degeneration.

In this paper: we developed three image enhancement techniques which are *Image Cartoonization, Edge Overlaying *and *Tinted Reduced Outlined Nature(TRON) *algorithms, which we have tested on both patients in trials and using our model. Image Cartoonization has previously been described in the image processing community [21,22]; here we describe its first use on patients with retinal degenerations. In the case of edge overlay, we have described an enhancement of this technique to improve segmentation of key features and removal of unnecessary ones. Finally, we present the *TRON *algorithm and its use in patients, which we believe will have advantages over edge only images as it maintains chromatic information.

## Methods

### A) Image enhancement algorithms

Human vision has its highest resolution with best visual acuity located at the fovea in the central macula much of the spatial processing of the visual cortex is designated to these regions. Patients with degeneration of the fovea and macula perceive an extreme blurred vision or a scotoma. In image processing terminology, there is a loss in the high frequency components of visual information. In addition, low contrast images can be particularly problematic. In order to improve this, our intention is to enhance the key features in the scene so as to enhance the effective contrast of the key features. As transferability to portable processing platforms is important, we have not attempted any form of saliency. Instead we use processing functions similar to those carried out by the retina and lower levels of the visual cortex which can be implemented on power efficient portable processing platforms [20,23].

#### TRON Algorithm

Low vision patients need a tool that can assist them in detecting moving objects normally without any delay or blurring effect. The *Tinted Reduced Outlined Nature(TRON)*, an algorithm which creates an edge-like image but maintains some chromatic content of the visual scene, aims to increase the contrast between objects by highlighting the edges of the moving objects and the edges between to distinguish objects while suppressing the other homogeneous pixels in the scene. It is performed in three steps:

1) *Simplification of the scene, using anisotropic filtering.*

2) *Extraction of the significant spatial derivatives, using a hierarchy method.*

3) *Boosting the original scene using the simplified spatial derivatives.*

Image simplification is an important step before performing spatial derivatives (edge extraction) so as not to extract high frequency noise and textures [24]. Gaussian filtering is a commonly used kernel for this purpose [25]. While it is effective at noise removal, it removes high frequency information, thus blurring the edges of the significant object boundaries.

Median filtering can be used to remove speckle noise. It is applied uniformly across an image, smoothing all pixels which appear to be considerably different to their neighbours. Thus while, it is very effective in the elimination of speckle noise, it is often at the expense of a slight blurring of the scene [25].

We therefore use a non-linear anisotropic smoothing technique to eliminate noise and low importance textures, while avoiding smoothing across object boundaries. It is an iterative process which progressively smoothes the image while maintaining the significant edges by reducing the diffusivity at those locations having a larger likelihood to be edges [26]. The process is defined as follows:(1)

I_t_(x) denotes the image intensity at position *x *and time *t*(I_0_(x) is the image at time t = 0 which is the original); ∇ is the gradient operator, and *div *is the divergence operator; c(x) is the diffusion coefficient (c(x) approaches 0 near edges, whereas it approaches 1 in homogeneous regions). The equivalent equation in the discrete domain is:(2)

Where n denotes the iteration number, Δt is the time step (it controls the accuracy and the speed of the smoothing) and ∇I_H_, ∇I_V_, ∇I_D1_, ∇I_D2 _represents the gradient in four directions.

The diffusion coefficient is then calculated from the following equation.(3)

After simplification, the next step is to obtain the gradient map. We use an algorithm described previously by Fleck [27] which based on a modified Canny filter [28]. Briefly, simple masks [-1, 0, 1] are used to compute the first derivative in four directions: *H *(horizontal), *V *(vertical), *D1*, and *D2 *(diagonal). The *X *and *Y *gradients are then computed by projecting the diagonal differences on both axes.(4)(5)

The amplitude of the gradient is:(6)

As simple high frequency (small kernel) derivatives of this form can be lossy in their boundary detection, we use a multi-scale pyramidal approach to obtain lower frequency (large kernel) derivatives. This is the equivalent of using multiple higher order kernels, but is more efficient in processing terms [29].

The final stage in the *TRON *algorithm to rescale the original image according to a weighting function *W *based on the gradient map. The gradient map is normalized to a fractional dynamic range between 0:1. We then define a threshold value *K *below which all the pixels will be raised to *K*. The original image is multiplied by the weighting function as given by:(7)

**Figure 1:**
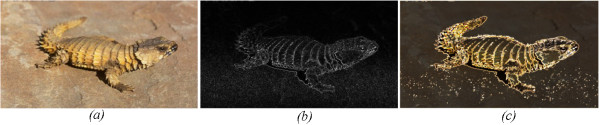
**TRON algorithm and the effect of image smoothing**. A low contrast image (a) and its first order gradient image using 3x3 Sobel kernel (b) compared to the TRON algorithm with the threshold value K set to 0.1 (c).

Figure 1(c), Shows the outcome of this algorithm compared to the basic edge detection from a first order derivative shown in Figure 1(b). The advantages of this technique over edge only images, is that it is more robust against noise and textures, and it maintains some of the chromatic information of the visual scene. By controlling the threshold *K *value we can increase or decrease the color information.

#### Cartoonization Algorithm

Image Cartoonization is a technique used to create stylized images that facilitate viewer recognition of the shapes by reducing visual clutters such as shadows and textures details [21,22]. This method improves the contrast of visually important features, by simplifying and reducing contrast in low-contrast regions and artificially increasing contrast in higher contrast regions. Our version of the algorithm has four main steps;

1) *Simplification of the image with anisotropic filtering*

2) *Calculating the spatial derivatives of the image*

3) *Quantization of the colors of the simplified image to create cartoon like images*

4) *Combining the quantized image with the negative of the gradient map*

The Algorithm starts by smoothing the original image using the above anisotropic diffusion filter as described in equations (1) to (3), above. The anisotropic diffusion is applied to the color image by converting it to the YCbCr color space [25], after that the Y (the intensity channel) is diffused. Then the YCbCr image is converted back to the RGB format. The gradient image calculated as given in equations (6) above, and normalized between 0 and 1. We then define two threshold values, *τ*_*min*_, *τ*_*max *_and we set all pixels of the normalized gradient image below *τ*_*min *_to 0 and all the pixels above *τ*_*max *_are set to 1.

To make paint-like effect on the image we quantize the luminance Y channel of the color image into bins:(8)

Q(x) is the quantized image, Δq is the bin width, q(x)_nearest _is the closest bin color to the current pixel *f*(*x*) and φ_q _is a matrix used to control the sharpness transition between one bin to another. The full description of this method has been described previously by Winnemoller et al [30] and is presented in more detail in the Appendix.

**Figure 2:**
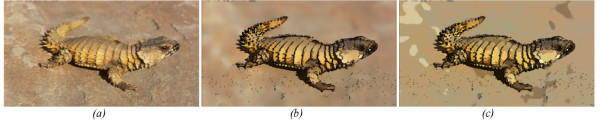
**Image Chartoonization**. A low contrast image (a) the cartoon image without color quantization (b) and the cartoon image with color quantization level set to 4(c).

To increase the visual distinctiveness of high contrast regions in the image we combined the negative of the corresponding extracted spatial derivatives described in equation (6) above. This negative gradient map overlay gives a notable edge enhancement, as can be seen in Figure 2. Figure 2(b) shows the *cartoonized *image without color quantization and Figure 2(c) shows the Cartoonization with the color quantization effect.

#### Edge Overlaying Algorithms

The edge overlay algorithms use the same mathematics as those previously described. Here, we recolor and overlay gradient map onto either the original image, or a simplified version of the original image. Thus contrast should be improved compared to the original.

**Figure 3:**
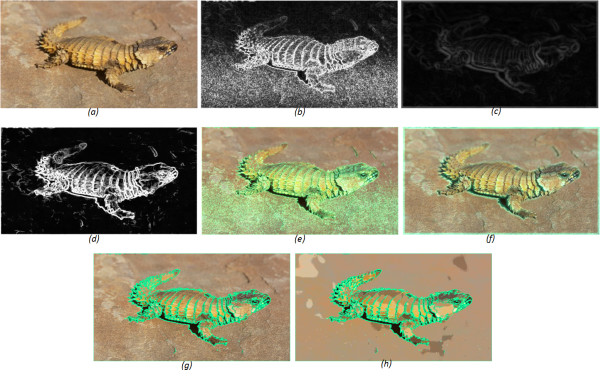
**Edge overlaying algorithm**. A low contrast image (a), (b) shows the extracted edges without applying any presmoothing filter edges and when smoothing using simple Gaussian (20 × 20 kernel size) and anisotropic diffusion filters in (c) and (d), respectively. (e-g) are the edge overlaid images when superimposed by edges extracted from the original image, simplified image by Gaussian and anisotropic diffusion filters, respectively. (h) is the overlaid on the cartoon image.

Wolffsohn et al. [8] previously tested a similar enhancement algorithm on visual impaired patients while watching television. The difference here is that Wolffsohn extracted the contour map with and without Gaussian smoothing. Thus, with smoothing, the image is slightly blurred compared to anisotropic simplification, and without results in the highlighting of many unwanted gradients as shown in Figure 3(b-d). Additionally, the Wolffsohn algorithm only used a 3 × 3 kernel, which makes it difficult to highlight the relevant contours over the irrelevant ones. In this paper we apply a simplification preprocessing step, as described in equations (1) to (3) above, to extract only the significant spatial derivatives. Additionally, we use a pyramidal approach to obtain the spatial derivatives across a range of spatial frequencies. Figure 3(e-f) shows the outcomes of the edge overlaying on the original image without smoothing and with Gaussian smoothing, respectively. Figure 3(g-h) shows the overlaying on the original and cartoon images when smoothing the image using the anisotropic diffusion filter.

### B) Degenerate retina model

There has been considerable previous work on modeling the human retina dating from Hubel and Wiesel [31]. The majority of this literature focuses on physiological aspects of the retinal function [32]. Other objectives include models for retinomorphic imaging systems which aim to mimic the human eye [33], and models for electronic retinal prostheses [34]. The majority of these models have been focused on modeling the normal retina rather than determining how information is distorted in the case of retinal degeneration.

Our model focuses on the centre surround spatial processing function of the retina. We reconstruct the chromatic and achromatic spatial information pathways being sent to the visual cortex. We can then reconstruct these for assessment of the visual information content.

The human retina is composed of several layers, organized in a highly structured network that extracts and pre-processes visual information from the image projected upon it [35,36]. Visual perception starts with image capture by the rod (achromatic, scotopic photoreceptors) and cones (chromatic - photopic photoreceptors). Our model ignores the difference between scotopic and photopic ranges as most imaging systems can only record with 8-bits of dynamic range and generally perform poorly at low light. We therefore separate the image matrix into achromatic (rod), blue (s-cone), green (m-cone), and red (L-cone) and yellow (for convenient opponent processing).

The centre surround spatial processing function in the retina results from the arrangement of the bipolar cells in particular and their connectivity to the retinal ganglion cells. The Horizontal cells perform smoothing and automatic gain control, the former can be performed through simple Gaussian filtering and the latter through histogram equalization. The amacrine cells are active in the achromatic and temporal processing, though we do not implement the latter as for this work we are investigating still images. The retina has two main visual pathways which transmit visual information to the visual cortex:

• The parvocellular pathway (P), which is responsible for transmission of chromatic spatial features, and is the dominant pathway from the central vision.

• The magnocellular pathway (M), which is responsible for achromatic and low-light spatio-temporal feature detection and is dominant in the peripheral vision.

**Figure 4:**
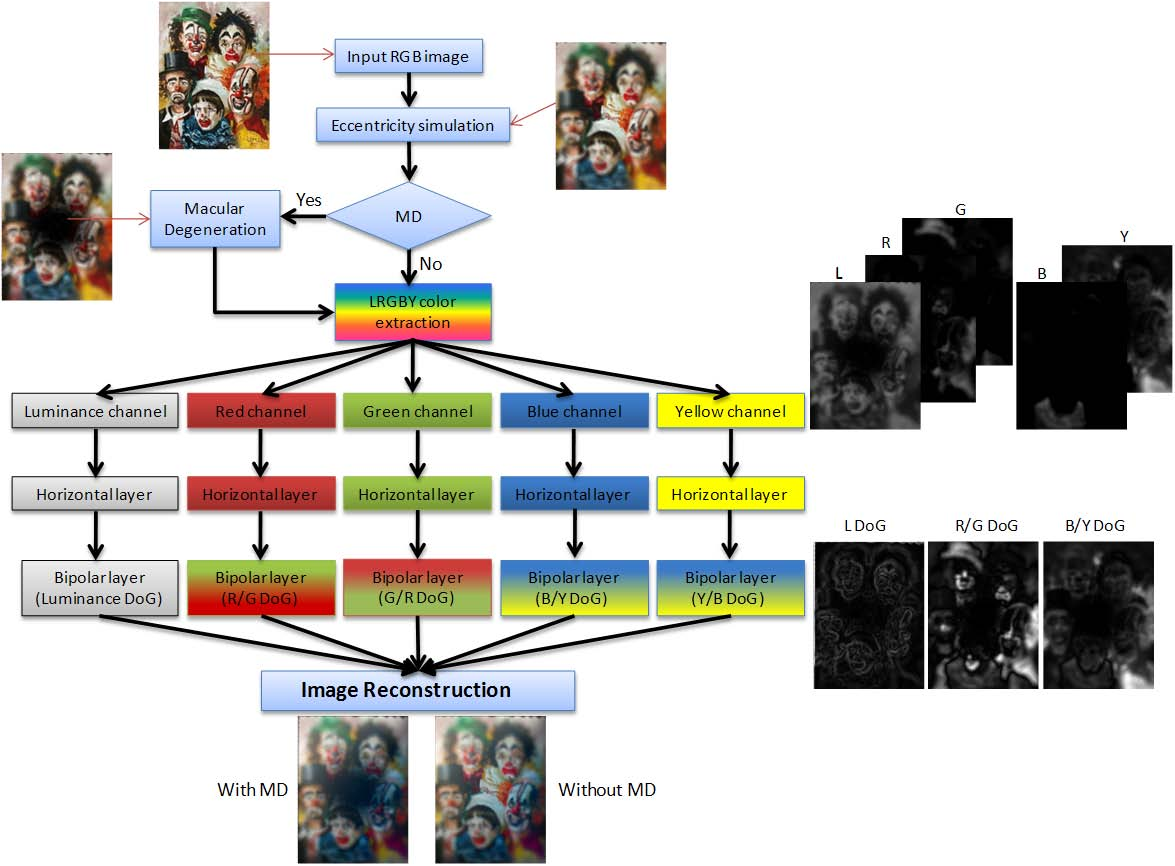
**Retinal model structure**. Structure of the normal retina model (simulating the foveal vision and the functions of the OPL) firstly by simulating the foveated vision using a multi-scale resolution sampling approach. Pixels in the fovea region are set to 1:1 from the input image while peripheral pixels are blurred by a Gaussian function with exponentially growing kernel size with radial distance from the fovea. The macular degeneration block is used here to simulate the degenerated photoreceptors. Then color is separated into four channels; Luminance, Red, Green, Blue and Yellow to be used in simulating the color opponent channels. The processed image is then reconstructed in the reconstruction module (three channels are reconstructed here; the Luminance, R/G and B/Y channels). Images are shown beside each stage for illustration.

In this work we are interested in the effect of spatial feature enhancement. Thus we model the P pathway and the non-temporal processing aspects of the M pathway. Our model is constructed from a linear combination of a set of spatial filters applied to the chromatic and achromatic color channels of the image matrix. Figure 4, shows the structure of our model. Our model represents the main processing layers of the retina, but we do not account for spike coding effects.

#### A. Simulating foveal vision

To reduce the amount of information passing through the optical nerve to the visual cortex region, the visual system of primates has a space-variant nature where the resolution is high in the fovea and gradually decreases towards the periphery of the visual field. Effective vision is possible due to rapid scanning (saccades) of the eye across the vision scene. By this method it is possible to achieve very high resolution via the fovea, while maintaining a rapid wide field of vision. To simulate this sampling behaviour we use a multi-scale resolution sampling methods [37,38], by dividing the image into two regions; fovea and periphery. The model has a 1:1 ratio of pixels in the fovea. The peripheral region is divided into concentric rings of equal width, which equivalent to one pixel. Each ring is blurred by a Gaussian function with kernel size growing exponentially with radial distance from the fovea. These variations with eccentricity represent the increasing size of retinal receptive fields with distance from the fovea.

We assume that the number of pixels in the input image approximates the number of cones sampling the retinal image. The number of pixels representing the fovea region in the input image is calculated based on the biological size of the fovea with respect to the retina. These dimensions are 1 mm and 42 mm respectively [35]. Thus, for an image size of 800 × 800, the number of pixels representing the fovea will approximately be 20 × 20. The foveal output image will be:(9)(10)

Where *r *is the radial distance of the pixel *(x, y) *from the centre of the input image, and *G*_*σ*_(*x*, *y*) is a two dimensional Gaussian averaging filter with a standard deviation *σ *equal to log(r). Figure 4, shows the output of applying the eccentricity simulation on an input image.

#### B. Color separation

The next stage in the model after simulating the foveal-peripheral vision is to account for the color separation in the retina. Input images are 2D matrices, with RGB components. In contrast, the chromatic information in the human retina is encoded into two color opponent channels; *green-red *and *blue-yellow*, and one achromatic channel. We therefore convert to a *LG*_*R*_*B*_*Y *_color space [25].

The *L *channel represents absolute luminance and extends from 0 (black) to 100 (white). The other two channels *G*_*R *_and *B*_*Y *_represent the greenness-redness and the blueness-yellowness color opponents respectively. Negative values of *G*_*R *_indicate green while positive values indicate magenta; similarly, *B*_*Y *_negative values indicate blue and positive values indicate yellow. Pixels for which *G*_*R *_= *B*_*Y *_= *0 *are achromatic and thus the *L *channel represents the achromatic scale of grays from black to white.

#### C. Horizontal layer

The horizontal cells serve as a negative feedback gain control on cone cells, adapting the reduction of glutamate release to increasing illumination. As the 8-bit dynamic range of most jpeg images is small, we consider the variation in illumination small, and thus we did not consider gain control in this model, although histogram equalization can be used to ensure optimal use of the 8-bit intensity range.

There are three types of the horizontal cells; HI (achromatic), HII and HIII (chromatic) cells. These cells have direct electrical synapses with each other and provide inhibitory feedback to the photoreceptors, with receptive field increasing towards the periphery. They are absent in the fovea. The horizontal cell function can be modeled with a diffusion process which results in a Gaussian-weighted spatial averaging of the cone inputs over the cell's RF.

The output of the horizontal cell can be obtained by convolving the cone output with an average Gaussian filter.(11)

Where *L, R, G, B *and *Y *represent the five separated channels: Luminance, Red, Green, Blue and Yellow.

#### D. Bipolar layer

Bipolar cells receive their inputs predominantly from the cones with some inhibitory feedback from the horizontal cells. ON bipolar cells depolarize with decreasing glutamate (increasing photo response) from the connecting photoreceptors, whereas OFF bipolar cells hyperpolarize. The synapses of surrounding ON and OFF bipolar cells to the retinal ganglion cells generate the centre-surround processing phenomena. In mammals, the ratio of the centre diameter field to the surround diameter one is range between 1:10 [39].

The centre-surround characteristics of the bipolar cells can be modeled in mathematical form as a difference of two Gaussian low pass filters (DoG). The surround filter, is more low-pass than the centre one. The DoG output to the retinal ganglion cells can be mathematically described as follows:(12)

*σ*_*s *_and *σ*_*c *_are the surround and centre standard deviation of the Gaussian filter. The ratio between the surround sigma to the centre one is considered to be 1:2, which give a reasonable agreement with the physiologically measured value [40]. Using this ratio value results in a receptive field diameter of the surround larger than the centre diameter by 5 to 6 times.

In the retina, centre surround processing is carried out for Red-centre/Green-surround, Green-centre/Red-surround, Blue-centre/Yellow-surround (parvocellular pathway) and achromatic ON-OFF centre-surround (magnocellular pathway). In this model we calculated five centre-surround signals as following:(13)

The size of the surround Gaussian kernel is set to 5 times larger than the size of centre kernel in each ON/OFF channel. Although there is no Yellow-centre/Blue-surround processing in the retina, we have included it here for purposes of processing symmetry.

#### E. Image reconstruction

Reconstruction can be achieved by reversing the processing operations carried out in the three retina layers. The output of DoG process of the bipolar cells can be considered as a spatial derivative of the achromatic, R/G and B/Y channels. Given this derivative (gradient) *G *for each channel, our task is to reconstruct an image *I *whose gradient ∇*I *is very similar to *G*. To achieve this, we can solve the equation ∇*I *= *G*. However, since the gradient image is a modified one from the actual gradients of the *L, G_R _*and *B*_*Y *_channels of the *LG*_*R *_*B*_*Y *_image, the resulting gradient field *G *= [*G*_*x*_, *G*_*y*_] may not be integrable [41]. To overcome this situation, we have to find a suitable function *I*, whose gradient should be very close to *G *using the least square error approach by searching the space of all 2D potential functions, that is, to minimize the following integral in 2D space:(14)

Where(15)

According to the Variational Principle, a function F that minimizes the integral must satisfy the Euler-Lagrange equation:(16)

Then we can drive a 2D Poisson equation:(17)

Where ∇^2 ^is the Laplacian operator and ∇·*G *is the divergence of the gradient *G*. There are different methods to solve the Poisson equation; such as finite difference methods, finite element methods and spectral methods. The fastest method is to solve it by using the *fast Poisson solver *method, which uses the *fast Fourier transform *to invert the Laplacian operator [42]. Figure 4, shows the result of a reconstructed image by solving the Poisson equations for the three opponent channels; Luminance, R_G _and B_Y _channels.

#### F. Macular degeneration simulation

The model described above simulates a normal retina. Degeneration can be implemented by turning off photoreceptors after the foveal simulation (eccentricity simulation) but before the color separation. We can thus create scotomata similar to that found in AMD patients.

The degeneration process starts by generating a binary mask that simulates these lesions on the scotoma region. The function that generates this mask takes three parameters; the location of the fovea with respect to the whole image (this refers to the area in the image where the person is fixating on it), the size of the degenerated area relative to the macula size, and the degree of degeneration.(18)

**Figure 5:**
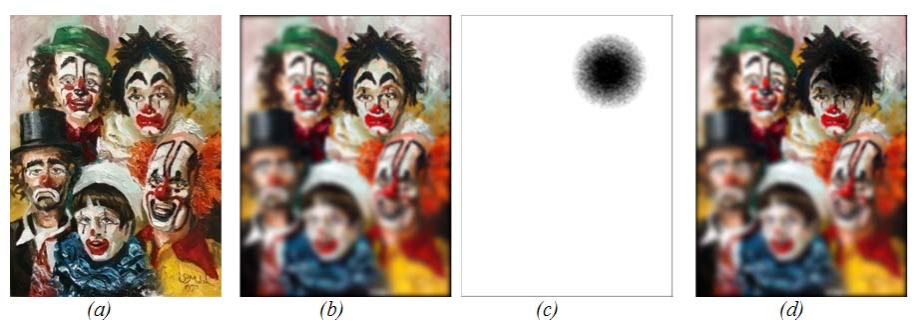
**Simulating scotoma (photoreceptor loss)**. The macular degeneration mask simulated in the mode: a) is the original image; b) is the foveated image with fovea focusing to the upper right part of the image; c) is the mask output that simulates retinal deterioration in the macula. Black pixels represent regions of photoreceptor loss; white pixels correspond to responsive regions of the normal photoreceptor mosaic. In this example, the black pixels cover a total of 100% of the macula region; d) is the degenerated image.

Where *M *is the mask output, *X*_*fovea*_, *Y*_*fovea *_is the *x *and *y *position of the fovea, *S *is the size of the defected region and *D *is the degree of degeneration. The output pixel of this mask is 0 or 1; 0 (black) pixels represent regions of photoreceptor loss [where *M(x, y) = 0*] and 1 (white) pixels correspond to responsive regions of the normal photoreceptor [where *M(x, y) = *1]. To simulate the blurring effect, the output is not simply set to zero. Rather, it has been ablated to simulate the diffusion of the photoreceptor loss by filling in the black spots with a Gaussian average of the pixels of the adjacent spots of healthy photoreceptors (pixels). Figure 5, shows the output of the mask for a fovea fixated to the top right part of an image with size of degeneration equivalent to the same size of the macula region which biologically equal to 6 mm (equivalent to 144 × 144 pixels for a 1008 × 800 image) [35].

The simulated degenerate retina is then the output of overlaying the foveal image with the degenerated mask.(19)

Figure 4, shows the degenerate retina model after adding the macular degeneration part and the output image of each block is shown beside each stage.

### C) Patient clinical trial protocol

27 patients were tested at the Oxford Eye hospital, John Radcliffe Hospital UK with approval from the Oxfordshire ethics committee. Of the patients; 9 were diagnosed with Retinitis Pigmentosa (RP), including 1 individual with Pseudoxanthoma Elasticum and 1 individual with Leber's Hereditary Optic Neuropathy. The remaining had macular pathologies, predominantly Stargardt's disease. The average visual acuity (VA) in the better eye in this cohort was 0.63 ± 0.07 (Range: -0.26: 1.14) and the average contrast sensitivity (CS) in the better eye was 1.22 ± 0.08 (Range: 0.15:1.65). The heterogeneity of the patient conditions was to allow us to broadly determine the effect of different severities and types of retinal degeneration on our enhancement methods.

Patients were presented with 25 sets of images and 4 sets of videos sequences. Images enhanced with our algorithms were randomly interspersed to even out the effect of memory. For each image, patients were asked to identify key scene features and were asked to rank different version of each image for both ease of major feature identification, and willingness to perceive images in this way in everyday life. In the case of the video sequences, these were placed next to each other and the patients asked to give viewing preference. The images and videos were projected to the patients using the Panasonic PT-AX200E projector with resolution of 1280 × 720 and maximum projection brightness (At a distance of 2 m from the projection wall) of 2000 Lumens, in a darkened room. The distance between the patient and the wall was kept to 1.5 m and the dimension of the projected screen was 110 cm width and 79 cm height so that the field of view was maintained at 40°.

## Results and Discussion

The results in this paper are divided into two sections; results obtained from testing the four image enhancement algorithms in our model, and results obtained from testing these algorithms on patients with retinal degeneration.

### Simulation results

The inputs into the model consist of original (unprocessed), and modified scenes and video sequences. The model was varied for various degrees of severity of retinal degeneration.

#### 1) Model simulation testing on still images

15 low contrast images were selected and enhanced with the four image enhancement algorithms. Then degenerated versions of these images were developed from the simulator. Model parameters included the size of the macular degeneration, strength of degeneration and fovea location. These were fixed for each image group to make an equal comparison of each algorithm. The outputs of the original and enhanced versions of each image were tested for perception efficacy by calculating the percentage of extracted edges on each image relative to the overall pixels on the image according to these three steps:

1) The gradient image was calculated as given in equations (6) above for each image.

2) Scaling the gradient image intensity between 0 and 255.

3) Summing up the intensities over the whole gradient image and dividing the result on the total number of pixels, according to this equation:(20)

Where G is the gradient image with dimension of M × N

**Figure 6:**
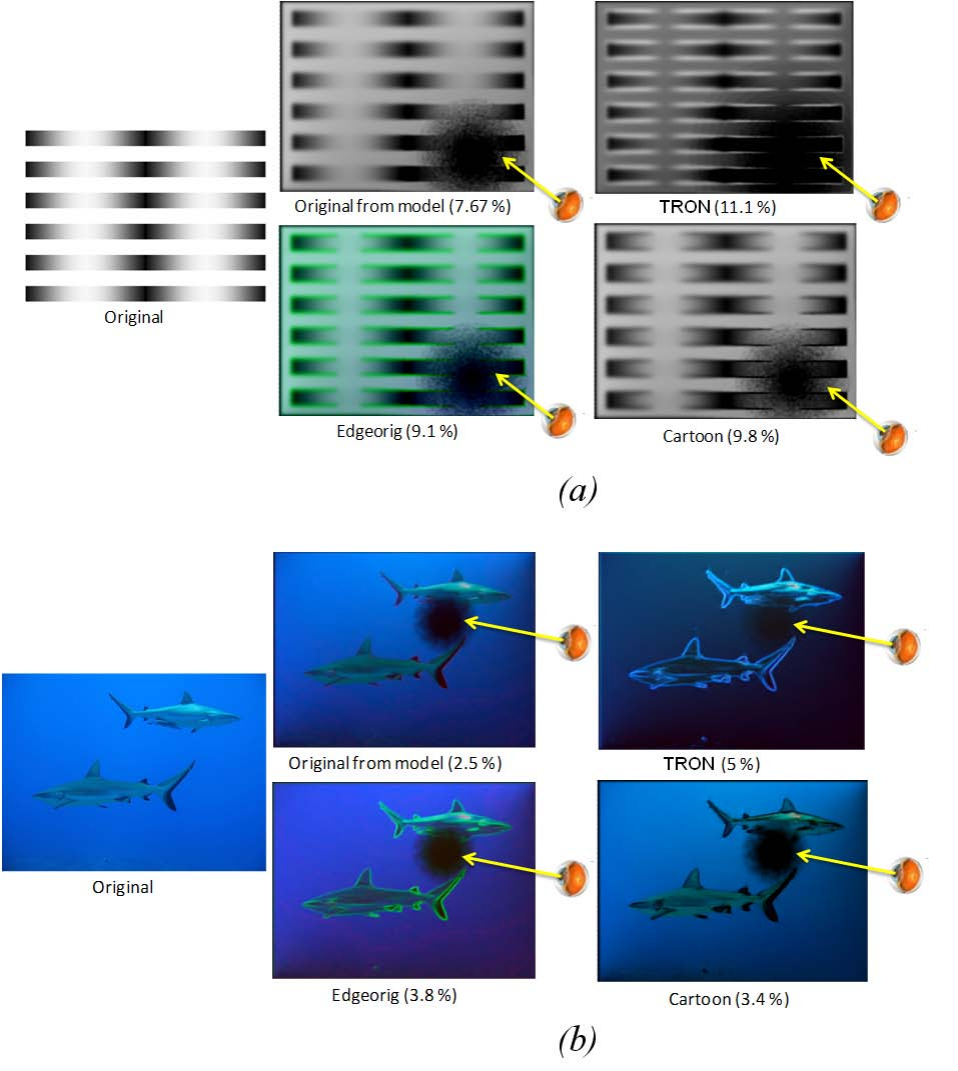
**The outcomes of the retinal model for each algorithm**. Testing the efficiency of image enhancement algorithms using the outputs from the retina model by calculating the percentage of extracted edges in the unprocessed and processed images. a) The macula here is looking to the lower right corner; b) The macula here is looking to the upper right corner. Percentage of edge detection (relative to the entire image) is shown under each image.

**Figure 7:**
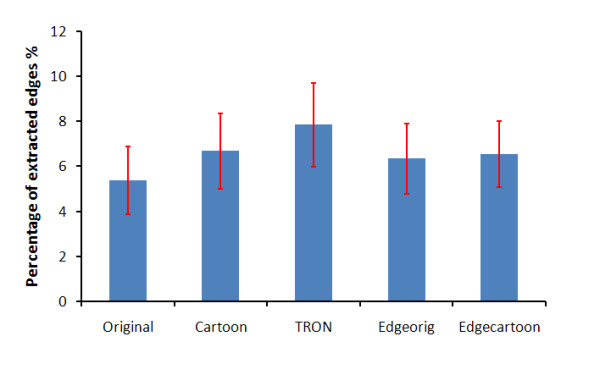
**The efficiency of image enhancement algorithms**. The efficiency of image enhancement algorithms using the outputs from the retina model by calculating the average percentage of extracted edges in the unprocessed and processed images over 6 different images.

Figure 6, shows the output of two images and enhanced versions thereof which have been passed through the degenerate retina model. The percentages of extracted edges are described under each image to show the efficacy of each algorithm. The percentage of extracted edges is higher in the images which have undergone prior visual enhancement processing. We do not present the output from the *edge on cartoon *algorithm in Figure 6 as it looks very similar to the *edge on original *processing function in this case. The average percentage of extracted edges over the 15 images for the unprocessed and processed images is shown in Figure 7.

From figure 7, it is highly significant that the TRON algorithm shows the highest performance in detecting and perceiving edges (P < 0.0176) followed by the *Cartoonization *(P < 0.0322) and *edge overlaying *(P < 0.0578).

**Figure 8:**
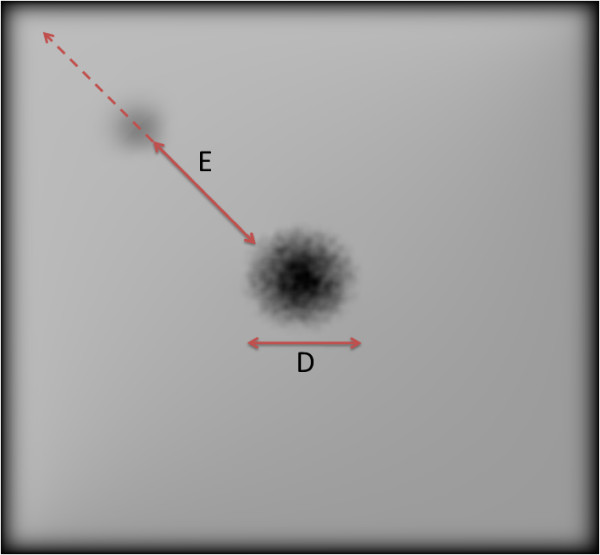
**Sample of the simulated image using the Pelli Robson's contrast sensitivity test**. The size of scotoma here is equivalent to the same size of macula. The contrast of object is 1.8 with eccentricity of 12.74 mm from the macula's centre. The arrows show the direction of eccentricity (E) from the macula's centre and the diameter (D) of the scotoma (which equals to the macula's diameter here).

**Figure 9:**
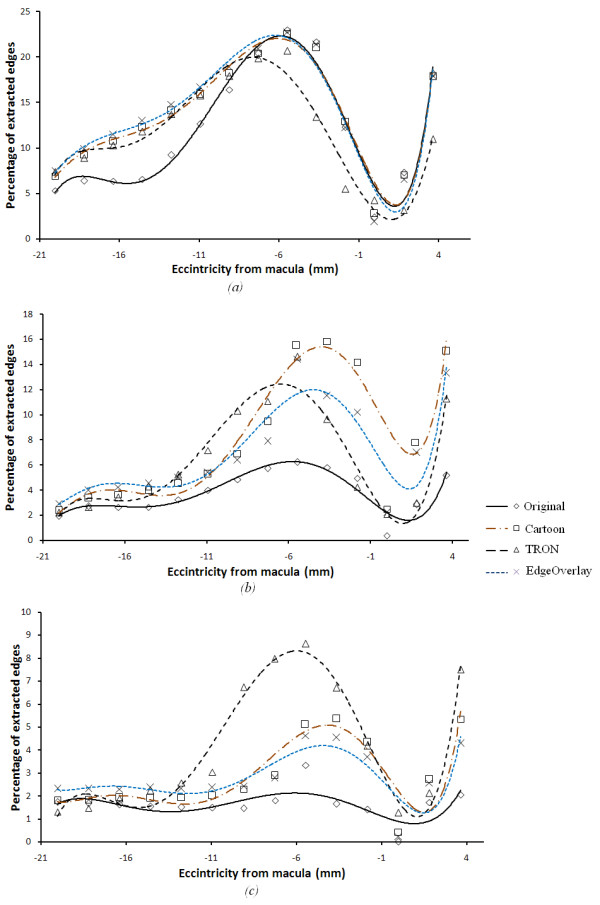
**Efficacy of enhancement algorithms on the simulated pelli Robson's images at different object contrasts**. (a-c) The efficacy of each algorithm compared to the original image on an image with object of 2.26, 1.66 and 0.75 contrasts, respectively, based on the Pelli Robson's method with respect to the eccentricity from the macula.

**Figure 10:**
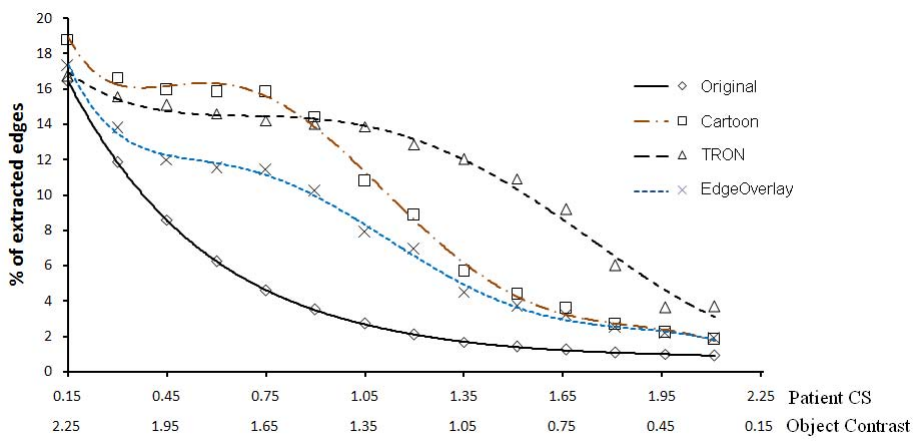
**The efficacy of enhancement algorithms on the simulated pelli Robson's images at eccentricity of 5.46 mm from the macula's centre**. The efficacy of each algorithm compared to the original image on an image with object at 5.46 mm from the macula's centre when changing the object contrast values.

In order to relate our simulator with the patient results, we tested our algorithms on the Pelli Robson's contrast sensitivity method [43] using our model for validation. Based on this method, we developed 16 (800 × 800) images with a white background and a gray box (of 44% the diameter of macula) with contrast ranging from 0 to 2.26. Each image is repeated 14 times to simulate the effect of eccentricity from the center of the macula. The eccentricity step was 30 pixels (equivalent to 0.26 of the macula's diameter). Degenerated versions of these images were developed from the simulator with a virtual scotoma of the same macula's size added to the center. Figure 8 shows a sample of image with contrast of 1.8 and eccentricity of 12.74 mm from the centre of the macula. Processed versions of these images have been generated using our three algorithms. The percentages of extracted edges have been calculated for the degenerated images before and after enhancement. Figure 9(a-c), shows the percentages of extracted edges for the unprocessed and processed image at contrasts of 2.26, 1.66 and 0.75 respectively. We can see that there is not much difference between the processed and unprocessed image of high contrast. The efficacy of the processed image over the unprocessed ones increases while decreasing the object's contrast as shown in Figure 9(b-c). This observation is clearly shown in Figure 10, which shows the percentage of extracted edges at eccentricity of 5.46 mm with respect to the patient CS (which opposite to the object's contrast here). To illustrate more what Figure 10 shows, the percentage of extracted edges at CS of 0.75 will be increased from 4.5 to 16.5. There is not much difference between the unprocessed and processed image neither at very low nor very high object contrasts. This is because the image processing algorithms we have used have difficulty in detecting very low contrast features. For high contrast objects, the enhancement algorithms will not add more detail to the object recognition process. However, the effort needed to recognize the processed image over the unprocessed is still decreased.

In order to determine more real-world effects of our algorithms, we examined the effect of our image enhancements on faced detection. This is an important function for daily interaction and is one of the key deficits affecting low vision patients. In computer vision field, face recognition algorithms initially perform the detection of a face, followed by identification of its distinguishing characteristics to perform identification [44]. Separate detection and recognition stages in the process of face perception in the human visual system has also been described in the neuropsychology literature [45]. For our purposes, as recognition involves much higher levels of cortical function, we use face detection as a primary test of the image enhancement algorithms.

Detecting faces requires the extraction of features that are common to all faces. In this paper we use two separate methods to test the efficiency of our algorithms in detecting faces; the first method is the Viola-Jones method [46], This is a featurebased algorithm, which attempts to detect the presence of certain facial features. It uses a cascade of increasingly complex filters, or feature detectors to improve performance to give a robust but quick detection. The first filter in the cascade consists of only two simple features, each composed of a few rectangular light and dark regions. Subsequent stages of filtering are performed only on regions scoring positive at any preceding stage. The Viola-Jones algorithm uses filter templates similar to the centre-surround phenomena in the human visual perception and is therefore additionally beneficial to this work. The second method is the Kienzle [47] appearance-based algorithm, which uses machine learning techniques to find relevant characteristics of face and non-face images. Then it builds discriminant function (i.e., decision surface, separating hyperplane, threshold function) to discriminate between these relevant characteristics of the faces and non-faces classes. Kienzle used the Support Vector Machines SVM classifier as the discrimination or the decision surface between the faces and non-faces classes [48]. SVM is a method to train polynomial function, neural networks, or radial basis function (RBF) classifiers. A full description of this method can be found in Kienzle paper.

**Figure 11:**
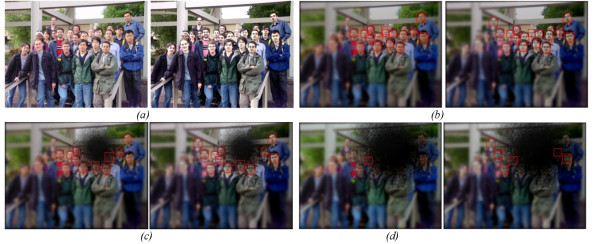
**Testing the ability of detecting faces in original and cartoonized image with different level of macular degeneration**. a) the original image to the left and the cartoonized version from it before the retina modelling, b) detection of faces on both the original image (left) and the cartoon image (right) without macular degeneration; only the foveal effect is shown here, c) with medium degeneration, and c) with severe degeneration.

We selected 14 images with different sizes of faces to give a sum total of 166 faces. Firstly, the original and processed images were degenerated by using our retina simulation model and then the output from the model fed to the Viola-Jones and Kienzle face detection algorithms. Each image underwent different levels of degeneration, starting from no degeneration to 4 times the biological macula size in increasing steps of 0.4, so in total we had 11 levels of degeneration for each image. Figure 11, shows the detected face rounded by squares in the original and *cartoonized *image with three levels of degeneration; no degeneration, medium degeneration and severe degeneration. Results show higher contrast around the faces of the *cartoonized *images.

**Figure 12:**
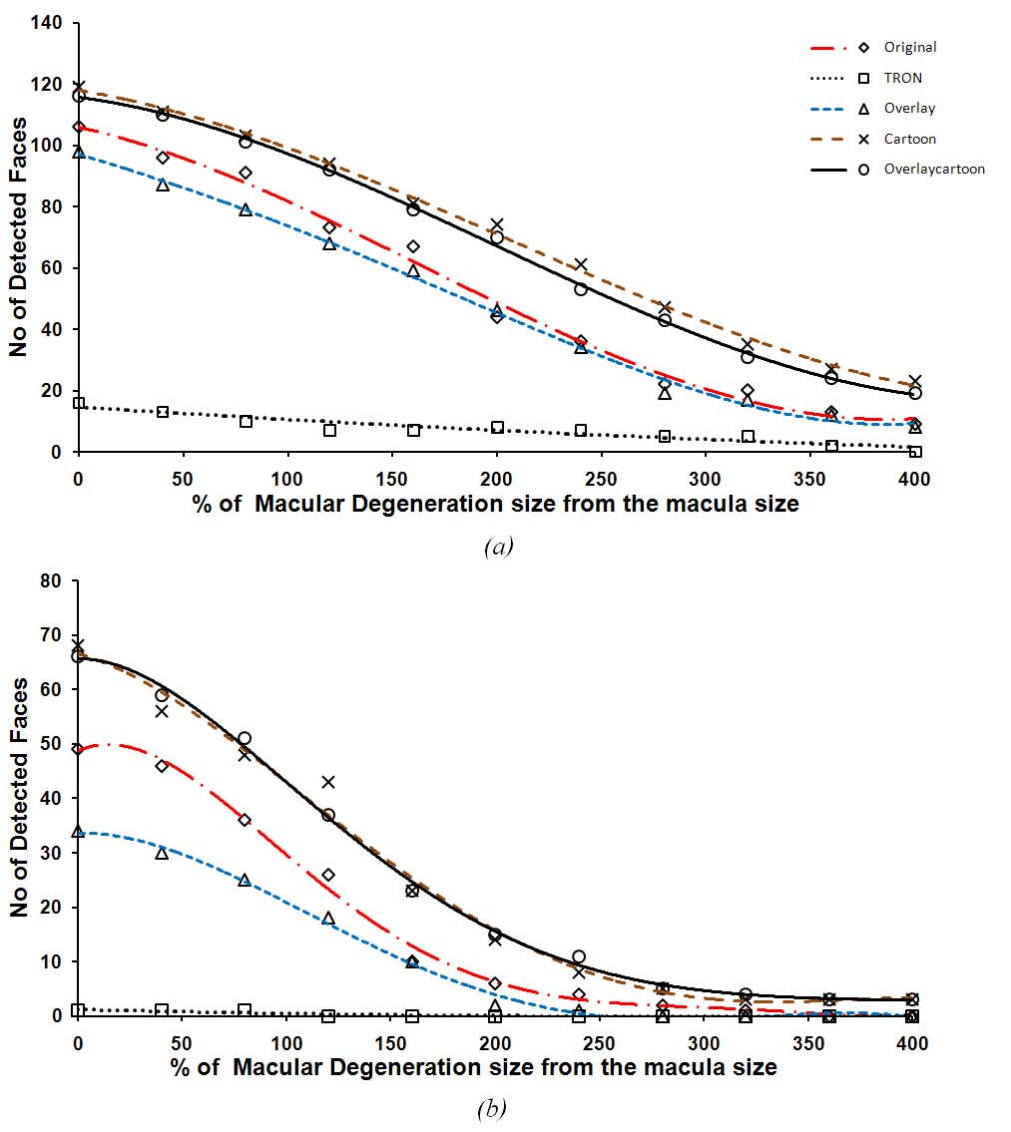
**The performance of face detection when image enhancement algorithms are used**. The number of detected faces over all the 14 images with different levels of macular degeneration for the original and the four image enhancement algorithms using the Viola-Jones face detection method (a) and Kienzle method (b).

Figure 12, shows the performance of each algorithm in enhancing the process of detecting faces compared to the original image using both the Viola-Jones and Kienzle algorithms, respectively. We can see that *Cartoonization *has the highest efficiency in detecting faces which was expected as *Cartoonization *enhances the contrast between boundaries while keeping the color information in the scene intact. *Edge overlaying *on cartoon images is less effective compared to *Cartoonization *in detecting faces when using the Viola-Jones algorithm and there is not much difference between it and the *Cartoonization *alone when using the Kienzle method. The original images were ranked as third and edge overlay on original was ranked last. We find that the TRON algorithm is not efficient in detecting faces. This is because the Viola-Jones used rectangular features (templates) that compare relative intensities of adjacent regions, and the Kienzle method works on the intensity level of the image pixels. In contrast, the TRON algorithm focuses mainly on enhancing the edges over the salient information in the scene. This suppresses most of the intensity information in the image and keeps only the boundaries between contrast regions.

#### 2) Testing dynamic scenes

**Figure 13:**
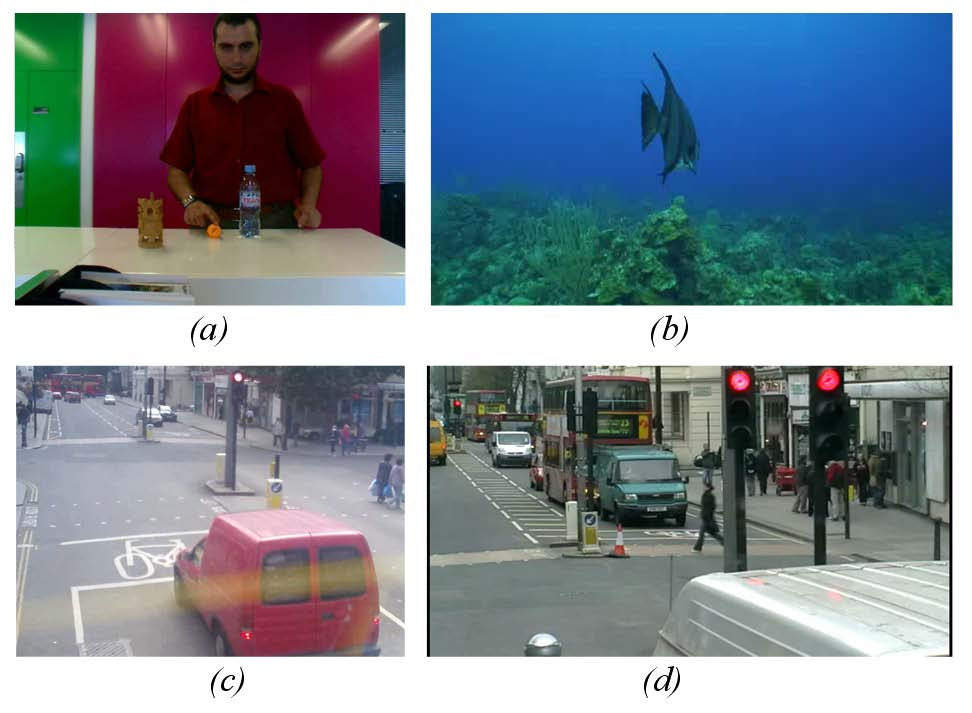
**Snapshots of the four videos used in the trial**. Snapshots of the four videos used in the trial. a) is an indoor video for a person doing different activity, b) an aquarium scene, c) and d) are two outdoor scenes for cars moving and people crossing the road.

Our purpose in this work is to develop algorithms to improve spatial feature recognition. In dynamic scenes, we hypothesize that enhancing the boundaries of moving objects will make their perception easier. We therefore tested our enhancement algorithms on 10 different video files, and determined efficacy on the basis of any improvement in motion detection of significant features. Snapshots for four of them are shown in Figure 13.

All of the ten files have been tested on our retinal model, and the first four of them have been tested on the patients. All the video files have frame rates of 25 fps and duration of 17 to 39 seconds. The files had been processed with our four image enhancement algorithms, and the degenerated versions of them (the unprocessed and processed files) were generated from our retina simulation model. The degeneration diameter was fixed in all the files to 2.5 times the size of the macula, to make a comparison. Motion detection between successive frames was detected for each file in 4 different levels to simulate different frame rates, e.g. motion was detected between 2, 3, 4 and 5 successive frames along the whole video length. To detect motion, we just do frame subtraction according to this equation:(21)

Then, the average motion detection from these different frame rates was used as the percentage of extracted edges as mentioned above in the method of measuring the percentage of perceived edges, and according to this equation:(22)

**Figure 14:**
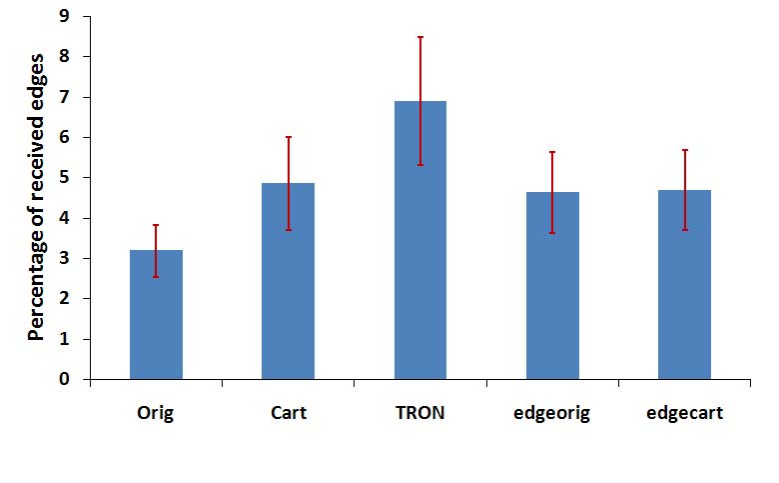
The efficiency of image enhancement algorithms in perceiving motion over 4 different movie files.

These percentage values of extracted edges were used to compare the efficiency of each algorithm for each movie file. Figure 14, shows the average percentage of extracted edges over the 10 files. We can see that the TRON algorithm shows the highest perception for edges (highest perception of detecting motion) (P < 0.0385).

### Patients' results

#### 1) Results from the still images

**Figure 15:**
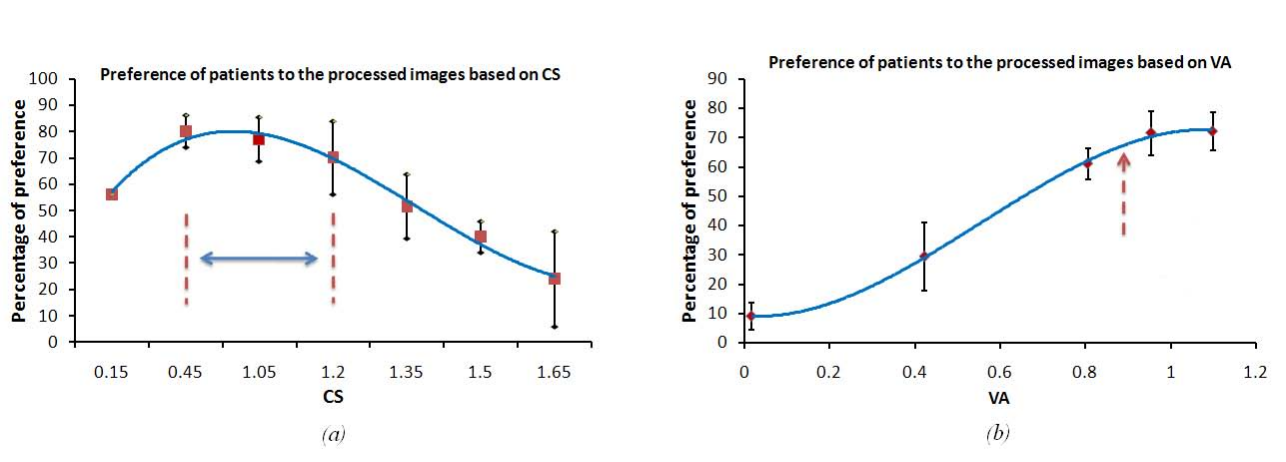
**The distribution of patient's preference to the processed images**. Based on their (a) contrast sensitivity CS, and (b) their visual acuity VA.

Patient data were divided into two groups: Group 1 comprised 11 patients who preferred to see more than 70% of the images in the processed version, and who had an average maximum contrast sensitivity of 1.02 ± 0.12, with an average VA of 0.8 ± 0.06 in their better eye. Group 2 comprised 16 patients who preferred unprocessed images had an average maximum contrast sensitivity of 1.35 ± 0.09, with an average VA of 0.52 ± 0.11 in their better eye. Figure 15, shows the distribution of all the patients preference for the processed images over the unprocessed images based on their CS and on their VA, respectively. From this figure we found that patients with CS 0.45 - 1.2 and VA greater than 0.9 showed a reasonable benefit from using our image enhancement algorithms.

**Figure 16:**
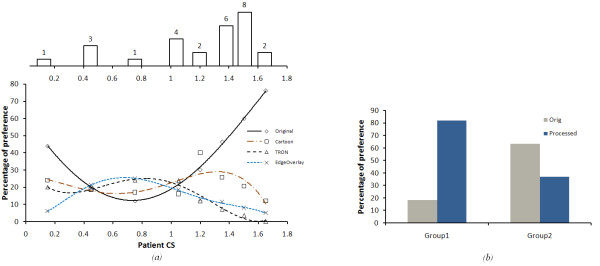
**The patient's preference to each algorithm over the unprocessed images (Static scene)**. (a)the patient's preference to the processed and unprocessed images according to their CS. the distribution of patients according to their CS is shown above the figure. (b)the preference of image enhancement over the original for both groups who preferred the 70% of images in the processed format and the others who preferred the enhanced images less than 70%.

Figure 16(a), is similar to what we have got from Figure 15. However, in this we show the distribution of preference for each algorithm over the whole patients CS range. Similar to the conclusion we have got from our model, which shown in figure 10, medium contrast scenes got a reasonable benefit from using the image enhancement algorithms. The distribution of cases according to the CS values is shown on the top of figure 16(a).

Figure 16(b), shows the patients' preference to the enhanced images relative to the original version, for the two groups of patients. Group 1 who preferred 70% of the images in the processed format and Group 2 who preferred the processed images less than 70%. For Group 1, we found that image *Cartoonization *was the most preferable for those patients, especially for images with low contrast, luminance and feature size. This was expected, as *Cartoonization *increases the contrast between the foreground objects and background. Furthermore, the added negative edges in the *Cartoonization *process added more contrast enhancement to the relevant features. Alternatively, edge overlay was preferred for scenes with high luminance and large-major features. One possible explanation is that high luminance can cause glaring and in that case, the differentiation between scenes objects will be difficult. Hence, by making a separation between foreground objects and background with different color can be more convenient for these patients. These results conclude that, *Cartoonization *and edge overlaying are the best for feature detection and recognition. *TRON *was least chosen, because it suppresses most of the natural and color information on the scene.

#### 2) Results from video

**Figure 17:**
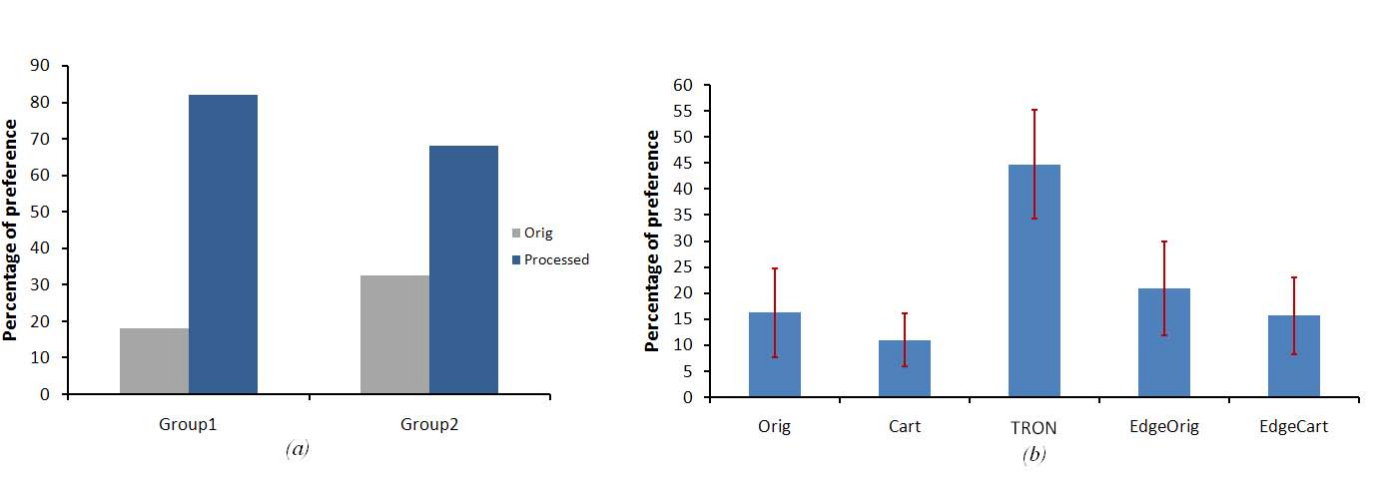
**The preference to video processed over the unprocessed**. (a)the preference of video enhancement over the original for both groups of patients. (b)The preference for patients of the first group with CS (0.45-1.2) and VA (> 0.9).

Figure 17(a), shows the results of patient preference in the motion detection tests for the two groups of patients. Patients from both groups preferred the processed videos over the unprocessed in enhancing the recognition and motion of objects. Figure 17(b), shows that the patients with CS between 0.4:1.2 and VA greater than 0.9 show a strong preference for the TRON algorithm. We can therefore conclude that TRON algorithm is the most useful in detecting objects which are moving (P < 0.0336). These results coincide with those from our model. This is because that *TRON *suppresses low frequency information and emphasizes high frequency information, so that it keeps very high contrast difference between moving objects and background.

From these observations we can conclude that presently there is no single algorithm which can be used for all the patients in all the circumstances. However, if implemented on a wearable augmented vision headset, patients could select the appropriate algorithm given the personal preference and visual situation. For example, *TRON *may be most appropriate for navigation. Alternatively *Cartoonization *and edge overlay may be most appropriate for watching television or more static scenes.

## Conclusions

In this article we have described three different image enhancement algorithms developed for patients suffering from retinal degenerative diseases. Additionally, we have presented an image processing model for retinal degeneration which we have used to evaluate the efficiency of these algorithms. The image processing model allows us to reconstruct the information stream towards the visual cortex and assess our algorithms using objective tests such as face detection. Results from this model show that TRON and *edge overlaying *algorithms are very useful in detecting spatial features in dynamic scenes and perceiving the edges of simple objects in static scenes. Image *Cartoonization *improves face detection. The same enhancement algorithms have also been tested on group of patients with primarily macular degenerations. When we analyzed the results from these patients we found that patients with CS range from 0.45 to 1.2 and VA greater than 0.9 derived the highest benefit from using these algorithms. This is highly consistent with the data from our model. Furthermore, the patient preference for the *Cartoonization *algorithm in static scenes and the TRON algorithm for dynamic scenes is also consistent with the findings from our model. In addition, to the identification of potential benefit of these two algorithms to the visually impaired, our testing methodology itself would be expected to be very useful in this field, as the ability to objectively determine efficacy of enhancement algorithms for those with low CS and VA will be beneficial for future studies. In the long run, we believe image enhancement algorithms such as that we present could perform the basis of the front end processing interface for retinal prosthesis [49] or new forms of visual assistive devices.

## Appendix: Short description of the Cartoonization algorithm

The Luminance channel is quantized into bins according to these equations:(23)(24)(25)

N is the number of bins which fixed in this paper to 8 bins. The numerator in equation (23) is set to 100 because the maximum value of the luminance channel is 100. *f*(*x*) is the pixel value of the luminance channel. INT means that the luminance channel will be rounded to the nearest integer value.

In equation (8), if *φ*_*q *_is fixed, then the transition sharpness is independent of the underlying image, creating many noticeable transitions in large smooth-shaded regions. To minimize these transitions, φ_q _is defined to be a function of gradient image. We allow hard bin boundaries only where the gradient is high. In low gradient regions, bin boundaries are spread out over a larger area.

According to Winnemoller et al [30], the sharpness range is set between [Λ_φ _Ω_φ_] and the gradient range to [τ_*min *_τ_*max*_]. The calculated gradient is clamped to [τ_*min *_τ_*max*_] and then *φ*_*q *_is generated by linearly mapping the clamped gradient map to [∇_φ _Ω_φ_].

We found that setting *τ*_*min *_= 0.1 and *τ*_*max *_= 0.4 of the normalized gradient image and the sharpness range to ∇_φ _= 3 and Ω_φ _= 25 give better edge enhancement.

## Competing interests

The authors declare that they have no competing interests.

## Authors' contributions

WA developed the algorithms, the retinal degeneration model, and performed the experimental work. MM and SD participated in the patients clinical trials. WA and PD performed the data analysis and manuscript writing. All authors read and approved the final manuscript
